# Congestion and renal function in patients with chronic heart failure

**DOI:** 10.1093/eschf/xvag126

**Published:** 2026-05-04

**Authors:** Ahmad Alsaeed, Htet Htet Ei Khin, Elisabetta Caiazzo, Moustafa I Morsy, Joe Cuthbert, Andrew L Clark, John G F Cleland, Pasquale Maffia, Pierpaolo Pellicori

**Affiliations:** School of Infection & Immunity, College of Medical, Veterinary and Life Sciences, University of Glasgow, Glasgow, UK; School of Cardiovascular & Metabolic Health, College of Medical, Veterinary and Life Sciences, University of Glasgow, Glasgow, UK; Department of Pharmacy, School of Medicine and Surgery, University of Naples Federico II, Naples, Italy; School of Infection & Immunity, College of Medical, Veterinary and Life Sciences, University of Glasgow, Glasgow, UK; Hull University Hospitals NHS Trust, Hull, UK; Hull University Hospitals NHS Trust, Hull, UK; School of Cardiovascular & Metabolic Health, College of Medical, Veterinary and Life Sciences, University of Glasgow, Glasgow, UK; School of Infection & Immunity, College of Medical, Veterinary and Life Sciences, University of Glasgow, Glasgow, UK; Department of Pharmacy, School of Medicine and Surgery, University of Naples Federico II, Naples, Italy; Africa-Europe CoRE in Non-Communicable Diseases & Multimorbidity, African Research Universities Alliance (ARUA) & The Guild of European Research-intensive Universities, Glasgow, UK; School of Cardiovascular & Metabolic Health, College of Medical, Veterinary and Life Sciences, University of Glasgow, Glasgow, UK

## Abstract

**Background:**

Cardiac and renal dysfunction often conspire to cause water and salt retention. We assessed the relation between renal function and congestion, both clinically and by ultrasound, in chronic heart failure (CHF).

**Method:**

At a routine clinic visit, patients with CHF were classified as clinically congested if they had a raised jugular venous pressure, pulmonary congestion, or peripheral oedema, regardless of severity, blind to a subsequent ultrasound assessment of congestion, including inferior vena cava (IVC) diameter, jugular vein diameter Valsalva/rest ratio (JVD-ratio), and lung B lines. Estimated glomerular filtration rate (eGFR) was calculated using the CKD-EPI 2021 equation.

**Results:**

Of 342 patients, eGFR was <30 in 9%, 30–44 in 18%, 45–59 in 26%, and ≥60 ml/min/1.73 m^2^ in 47% of the patients. Many patients had both clinical and ultrasound evidence of congestion, especially when eGFR was low (for each eGFR group, respectively: 51%, 30%, 39%, and 25%). Isolated ultrasound congestion was also common (19%, 29%, 34%, and 26%), but isolated clinical congestion was less so (11%, 11%, 4%, and 9%).

Congestion, especially when detected by both methods, was associated with higher NT-proBNP concentrations and a greater probability of heart failure (HF) hospitalization or death (adjusted HR: 2.16, 95% CI [1.25, 3.75]; *P* = .006 vs no congestion).

**Conclusion:**

Patients with CHF often have clinical evidence of congestion, confirmed by ultrasound, which is associated with a poor prognosis. Patients with a low eGFR are more likely to be congested. Whether ultrasound assessment of congestion can improve the management of patients with CHF requires more attention.

## Graphical Abstract

**Graphical Abstract:**
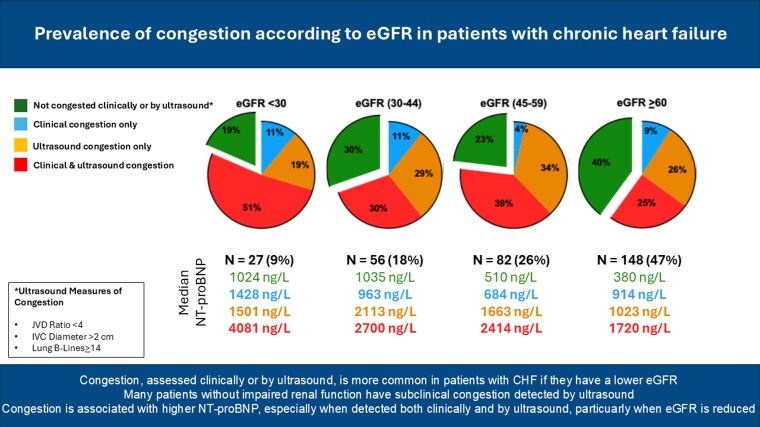
Prevalence of congestion according to estimated glomerular filtration rate (eGFR) in patients with chronic heart failure. 313 patients had complete data on ultrasound signs of congestion. With worsening renal function, there were greater degrees of congestion. Patients with both clinical and ultrasound evidence of congestion (in red) had the highest NT-proBNP. Abbreviations: CHF, chronic heart failure; IVC, inferior vena cava; JVD, jugular vein diameter.

## Background

Congestion due to renal retention of water and salt is a key feature of heart failure (HF) that is associated with more severe symptoms, worse quality of life, adverse cardiac remodelling, poor outcomes, and substantial health care costs.^1,2^

Many patients with chronic HF (CHF) have impaired renal function, with about half having an estimated glomerular filtration rate (eGFR) <60 ml/min/1.73 m^2^.^3^ Kidney and heart failure share common risk factors for their development and are closely linked pathophysiologically. A low renal perfusion has traditionally been considered a major contributor to renal dysfunction; however, more recent evidence suggests that elevated venous pressures also have deleterious effects on renal function in patients with CHF, exacerbating sodium and water retention and perpetuating a vicious cycle of congestion.^4,5^

Although congestion is central to HF pathophysiology and prognosis, its assessment is imprecise. HF guidelines strongly recommend that congestion should be treated with loop diuretics, but clinical manifestations of congestion are observer-dependent (both patient and health professional) and become obvious only when HF is advanced. Ultrasound assessment of the inferior vena cava (IVC), jugular vein, and pulmonary parenchyma may aid in identifying patients with CHF who are sub-clinically congested or quantify congestion with more precision. In other words, they help address this gap.^6^

In this study, we assessed the prevalence and severity of congestion, clinically and by ultrasound, in patients with CHF with different degrees of renal dysfunction and explored the relation between congestion and outcome. We hypothesized that renal dysfunction would be associated with a higher prevalence and greater severity of congestion, particularly when detected by ultrasound.

## Methods

We assessed consecutive patients with CHF during a routine clinic appointment over 12 months (between April 2016 and March 2017). All patients gave informed consent. The study conformed to the principles outlined in the Declaration of Helsinki and was approved by relevant ethical bodies. This study was embedded within a clinical service providing routine care to patients with an established diagnosis of HF; therefore, no specific inclusion or exclusion criteria were applied. Patients were assessed following a diagnosis of heart failure, which had been established based on elevated NT-proBNP levels in primary care or after a hospital admission for heart failure.

Patients underwent blood tests—including haematology, biochemistry profile, and N-terminal pro-B-type natriuretic peptide (NT-proBNP)—as well as electrocardiograms (ECG) and echocardiograms on the same day.

Clinical examination was performed before echocardiography. Systematic assessment included lungs (normal findings, presence of basal, mid-zone, or diffuse crackles), jugular vein (not visible, raised jugular venous pressure: 1–4 cm, or raised to earlobe), and legs (no peripheral oedema, oedema to ankles, below or above knees). Patients were classified as clinically congested if they had at least one sign of congestion, regardless of severity.^7^

Echocardiography was performed by a cardiologist experienced in echocardiography using a Vivid Seven (GE Healthcare, UK) system operating at 1.7–3.4 MHz, in accordance with international reccomandations^8^ The IVC diameter was measured 1–3 cm before it merged with the right atrium by placing the ultrasound probe in the subcostal area. The internal jugular vein was assessed with a linear, high-frequency probe by placing the patient in a reclining position with the head and neck elevated at 45°. We measured the ratio between the internal jugular vein’s maximum diameter during the Valsalva manoeuvre (performed by forcing expiration against a closed glottis) and that at rest (JVD ratio), as previously described.^9^ A total of 28 intercostal spaces were scanned (from the second to the fifth intercostal space on the right hemithorax and from the second to the fourth intercostal space on the left hemithorax, along the parasternal, midclavicular, anterior axillary, and midaxillary lines) to calculate the total number of lung B lines.^10^ Echocardiograms were stored and reviewed by a single experienced operator off-line.

Patients were classified as congested by ultrasound if they had an IVC diameter of >2 cm, a JVD ratio of <4, or a total number of B lines ≥14.^10^ The CKD-EPI 2021 formula was used to calculate eGFR.^11^ The cohort was stratified by different eGFR categories (<30, 30–44, 45–59, ≥60 mL/min/1.73 m^2^) according to Kidney Disease Improving Global Outcomes (KDIGO) criteria for chronic kidney disease.^12^

Categorical data are presented as numbers and percentages, while continuous data are shown as medians with the first and third quartiles. To compare categorical variables, χ2 or Fisher’s exact tests were used. For continuous variables, ANOVA was used when the data were normally distributed; otherwise, the Kruskal-Wallis test was used. A multivariable Cox proportional hazard regression model was used to assess the relation between congestion and the risk of HF hospitalization or death, using a limited number of variables known to be strongly associated with outcome in this population [age, sex, left ventricular ejection fraction (LVEF), eGFR (continuous), NYHA class, atrial fibrillation, diabetes, haemoglobin, use of loop diuretics, and mineralocorticoid receptor antagonists (MRA)] to prevent statistical over-fitting. Kaplan–Meier curves, censored at 2 years of follow-up, were used to illustrate the outcome. All analyses were conducted using R (4.4.2). Two-tailed *P* values < .05 were considered statistically significant.

## Results

Of 342 patients (median age 75 years), 67% were men, the median LVEF was 47 (35–55) %, and the median NT-proBNP was 568 (258–1606) ng/L for those in sinus rhythm and 1916 (1176–3501) ng/L for patients in atrial fibrillation (*n* = 164, 48%).

Patients with eGFR ≥60 mL/min/1.73 m^2^ were younger. With increasing renal dysfunction, haemoglobin was lower, NT-proBNP was higher, use and dose of loop diuretics were greater, and MRA were used less often (*Table 1*). Patients with more severe renal dysfunction had greater left atrial volume when indexed for body surface area (LAVI), worse right ventricular function measured by TAPSE, and were more likely to have mitral and tricuspid regurgitation. However, LVEF was similar regardless of eGFR (*Table 2*).

**Table 1:** Baseline characteristics, treatment, and blood test results for patients with chronic HF stratified by estimated glomerular filtration rate (eGFR)

Characteristics	eGFR <30 Median (IQR) 24 (22–25) N = 29^a^	eGFR (30–44) Median (IQR) 36 (33–41) N = 62^a^	eGFR (45–59) Median (IQR) 54 (48–56) N = 87^a^	eGFR ≥60 Median (IQR) 80 (69–91) N = 164^a^	*P-*value*
Age, years	77 (73, 83)	80 (74, 85)	77 (69, 83)	71 (63, 78)	**<**.**001**
Men	18 (62%)	40 (65%)	60 (69%)	111 (68%)	.88
DM	11 (38%)	16 (26%)	28 (32%)	44 (27%)	.52
BMI, kg/m^2^	29 (28, 33)	29 (25, 33)	28 (26, 34)	30 (25, 33)	.79
Smoker	5 (17%)	4 (6%)	7 (8%)	27 (16%)	.08
HTN	16 (55%)	37 (60%)	52 (60%)	83 (51%)	.45
IHD	16 (55%)	38 (61%)	42 (48%)	71 (43%)	.1
COPD	7 (24%)	7 (11%)	17 (20%)	35 (21%)	.33
HR, bpm	75 (68, 78)	67 (60, 75)	71 (65, 81)	70 (62, 80)	.42
SBP, mmHg	130 (119, 149)	136 (121, 154)	136 (120, 155)	136 (123, 156)	.93
DBP, mmHg	68 (60, 77)	71 (63, 78)	73 (65, 80)	76 (65, 84)	.13
Atrial fibrillation	15 (52%)	28 (45%)	47 (54%)	74 (45%)	.54
NYHA					.**008**
I	3 (10%)	5 (8%)	7 (8%)	35 (21%)	
II	15 (52%)	39 (63%)	53 (61%)	100 (61%)	
III	11 (38%)	18 (29%)	27 (31%)	29 (18%)	
Blood tests					
Hb, g/dL	12.1 (11.2, 13.6)	12.3 (11.5, 13.1)	13.2 (11.9, 14.1)	13.6 (12.6, 14.7)	**<**.**001**
Urea, mmol/L	19.7 (14.9, 22.8)	12.9 (10.3, 16.5)	9.6 (7.7, 11.5)	6.9 (5.2, 8.1)	NA
Creatinine, μmol/L	219 (189, 242)	153 (131, 171)	116 (105, 127)	81 (72, 95)	NA
Bilirubin, μmol/L	11 (7, 14)	11 (8, 16)	11 (8, 14)	11 (8, 14)	.98
ALT, U/L	16 (12, 21)	16 (12, 22)	16 (13, 21)	19 (15, 25)	**<**.**001**
ALP, U/L	108 (78, 128)	87 (68, 116)	83 (66, 101)	78 (65, 100)	.**02**
Albumin, g/L	35 (33, 38)	36 (34, 37)	36 (34, 38)	37 (35, 39)	**<**.**001**
NT-proBNP AF, ng/L	3570 (1240, 6972)	2615 (1769, 4524)	2299 (1511, 4813)	1422 (915, 2556)	**<**.**001**
NT-proBNP SR, ng/L	1465 (872, 3017)	1153 (504, 2476)	785 (300, 1990)	355 (175, 919)	**<**.**001**
Treatment					
Beta-blocker	22 (76%)	56 (90%)	70 (80%)	139 (85%)	.23
ACEi/ARBs	25 (86%)	50 (81%)	75 (86%)	140 (85%)	.79
MRA	8 (28%)	36 (58%)	47 (54%)	75 (46%)	.**03**
CRT	2 (6.9%)	6 (9.7%)	11 (13%)	9 (5.5%)	.23
Furosemide	27 (93%)	50 (81%)	74 (85%)	107 (65%)	**<.001**
20 mg/day	1 (3.4%)	4 (6.5%)	8 (9%)	20 (11%)	
40 mg/day	11 (38%)	20 (32%)	29 (34%)	55 (34%)	
≥40 mg/day	15 (52%)	26 (42%)	36 (42%)	32 (20%)	

Abbreviations: DM, diabetes mellitus; BMI, body mass index; HTN, hypertension; IHD, ischaemic heart disease; COPD, chronic obstructive pulmonary disease; SBP, systolic blood pressure; DBP, diastolic blood pressure; HR, heart rate; NYHA, New York heart association; BB, beta-blocker; ACEi, angiotensin-converting enzyme inhibitors; ARBs, angiotensin receptor blockers; MRA, Mineralocorticoid receptor antagonist; CRT, cardiac resynchronization therapy; Hb, haemoglobin; ALT, alanine transaminase; ALP, alkaline phosphatase; NT-proBNP, N-terminal pro b type natriuretic peptide; bpm, beats per minute; AF, atrial fibrillation; SR, sinus rhythm

^a^Median (Q1, Q3); n (%).

^*^Kruskal-Wallis rank sum test; Pearson’s χ2 test; Fisher’s exact test; One-way ANOVA; NA (Not applicable).

**Table 2:** Echocardiography

Characteristic	eGFR <30 Median (IQR) 24 (22–25) N = 29^a^	eGFR (30–44) Median (IQR) 36 (33–41) N = 62^a^	eGFR (45–59) Median (IQR) 54 (48–56) N = 87^a^	eGFR ≥60 Median (IQR) 80 (69–91) N = 164^a^	*P*-value*
LVEDV, mL	134 (105, 208)	148 (101, 175)	150 (112, 226)	139 (108, 190)	.24
LVEF, %	44 (36, 55)	49 (37, 58)	43 (30, 54)	49 (36, 55)	.07
LVEF >50%	12 (41%)	29 (47%)	28 (32%)	70 (43%)	.28
LAVi, mL/m^2^	46 (37, 58)	45 (32, 65)	48 (38, 61)	41 (32, 54)	.**006**
MR no/trivial	10 (34%)	28 (45%)	31 (36%)	93 (57%)	.**006**
MR mild	19 (66%)	28 (45%)	50 (57%)	66 (40%)	
MR moderate/severe	0 (0%)	6 (10%)	6 (7%)	5 (3%)	
TAPSE, cm	1.8 (1.4, 2.1)	1.9 (1.5, 2.3)	1.9 (1.4, 2.2)	2.0 (1.6, 2.4)	.**01**
TR, no/trivial	9 (31%)	33 (53%)	39 (45%)	100 (61%)	.**007**
TR, mild	17 (59%)	26 (42%)	44 (51%)	61 (37%)	
TR, moderate/severe	3 (10.0%)	3 (4.8%)	4 (4.6%)	3 (1.8%)	

Abbreviations: LVEDV, left ventricular end-diastolic volume; LVEF, left ventricular ejection fraction; LAVi, left atrial volume index; TAPSE, tricuspid annular plane systolic excursion; MR, mitral regurgitation; TR, tricuspid regurgitation

^a^Median (Q1, Q3); n (%).

^*^Kruskal-Wallis rank sum test; Pearson’s χ2 test; Fisher’s exact test; One-way ANOVA.

**Figure 1:**
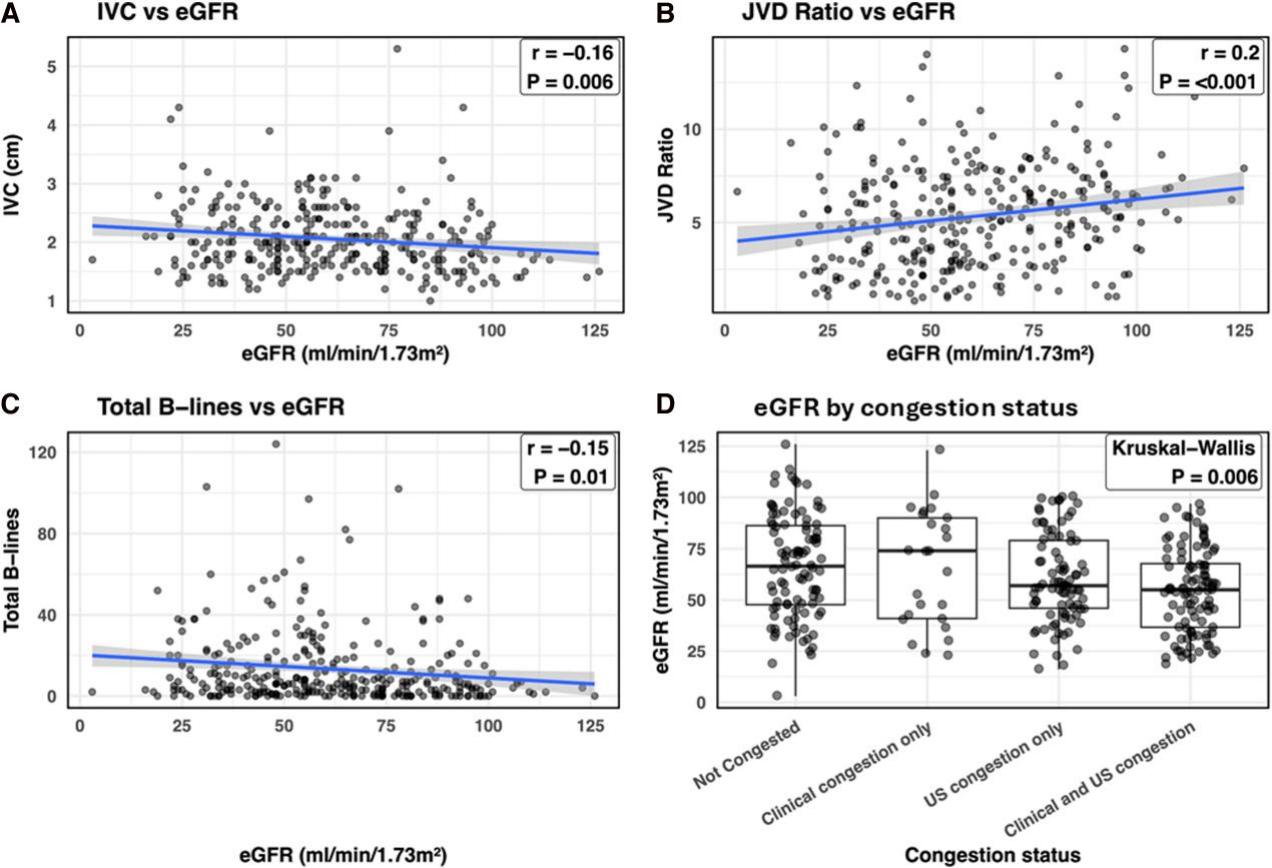
Scatter plots showing the relation between ultrasound signs of congestion (IVC diameter, JVD ratio, and total number of B lines) and eGFR (panels A, B, and C, respectively). Panel D (bottom right) shows box plots of eGFR across different congestion phenotypes. Patients with both clinical and ultrasound (US) congestion had the lowest median eGFR.

Of 342 patients enrolled, 313 had complete data on ultrasound signs of congestion; the inferior vena cava was not visualised in 7 patients, and JVD-ratio was not measured in 23 patients because the relevant ultrasound probe was not available. With worsening renal function, the presence and severity of congestion increased, both clinically and on ultrasound (*Table 3* and *graphical abstract*). However, there was only a weak relationship between eGFR and IVC diameter ((correlation coefficient (*P*)): (−0.16 (.006)); JVD-ratio (0.20 (<.001)); and B-lines, (−0.15 (.01)) (*Figure 1*).

**Table 3:** Congestion signs

Characteristic	eGFR <30 Median (IQR) 24 (22–25) N = 29^a^	eGFR (30–44) Median (IQR) 36 (33–41) N = 62^a^	eGFR (45–59) Median (IQR) 54 (48–56) N = 87^a^	eGFR ≥60 Median (IQR) 80 (69–91) N = 164^a^	*P*-value*
Peripheral Oedema					
Oedema, none	13 (45%)	39 (63%)	56 (64%)	117 (71%)	.**042**
Oedema, ankles	4 (14%)	10 (16%)	13 (15%)	23 (14%)	
Oedema, >ankles	12 (41%)	13 (21%)	18 (21%)	24 (15%)	
Lung Crackles					
Lung crackles, none	22 (76%)	54 (87%)	73 (84%)	153 (93%)	.**015**
Lung crackles, basal	7 (24%)	8 (13%)	14 (16%)	11 (7%)	
Jugular Venous Pressure					
JVP, not raised	19 (65%)	50 (81%)	65 (75%)	138 (84%)	.074
JVP, 1–4 cm	8 (28%)	8 (13%)	18 (21%)	21 (13%)	
JVP, to earlobe	2 (7%)	4 (6%)	4 (4%)	5 (3%)	
Congestion by Ultrasound					
JVD-ratio	3.1 (2.1, 6.6)	5.0 (3.0, 7.0)	3.9 (2.6, 6.7)	5.9 (4.2, 7.4)	.**005**
Total B-lines	8 (2, 20)	9 (2, 17)	9 (3, 29)	5 (1, 12)	.**001**
IVC diameter, cm	2.1 (1.7, 2.6)	2.0 (1.7, 2.4)	2.0 (1.8, 2.5)	1.8 (1.6, 2.3)	.09

Abbreviations: JVP, jugular vein pressure; JVD-ratio, Jugular venous diameter Valsalva to rest ratio; IVC, inferior vena cava diameter

^a^Median (Q1, Q3); n (%).

^*^Kruskal-Wallis rank sum test; Pearson’s χ2 test; Fisher’s exact test; one-way ANOVA.

**Figure 2:**
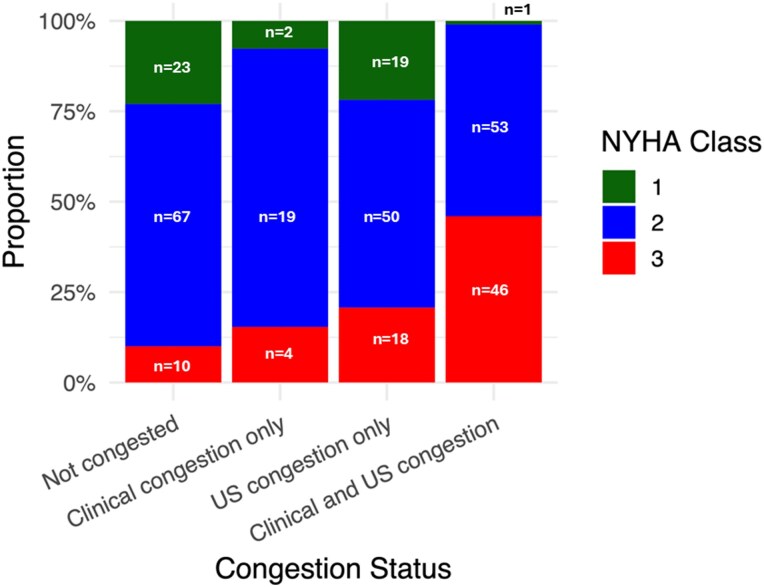
Congestion and symptoms. Patients with both clinical and ultrasound (US) evidence of congestion were more likely to have severe symptoms. ‘N’ indicates the number of patients in each group.

**Figure 3:**
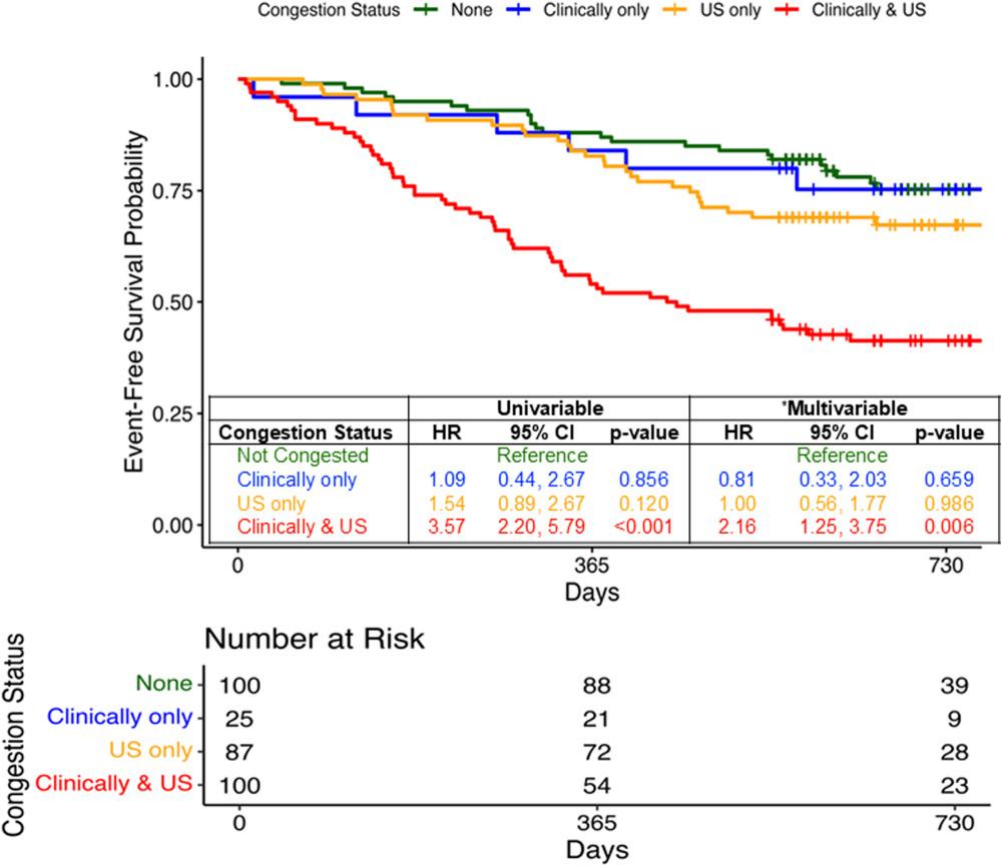
Congestion and outcome. Kaplan–Meier curves for the composite endpoint of heart failure hospitalization or death. Compared to those not congested, patients with concurrent clinical and ultrasound (US) congestion had a 2-fold greater risk of an adverse event (*adjusted for age, sex, left ventricular ejection fraction, estimated glomerular filtration rate, NYHA class, atrial fibrillation, diabetes mellitus, haemoglobin, use of loop diuretic and mineralocorticoid receptor antagonists: HR: 2.16, 95% CI 1.25–3.75; *P* = .006).

Over a median of 606 (373–754) days of follow-up, 117 patients with complete ultrasound data were hospitalized with heart failure or died. Congestion was associated with more severe symptoms (*Figure 2*), higher plasma NT-proBNP (Supplementary table S1), and, when detected clinically and confirmed by ultrasound, a higher probability of heart failure hospitalization or death (unadjusted hazard ratio [HR] 3.57, 95% confidence interval [CI] 2.20–5.79; *P* < .001; adjusted HR 2.16, 95% CI 1.25–3.75; *P* = .006) (*Figure 3*). Neither clinical congestion nor US congestion alone was associated with an adverse prognosis.

## Discussion

We found that in patients with CHF, the prevalence of congestion increases as eGFR decreases. We also found that ultrasound markers of congestion are more common than clinical congestion alone and that they are associated with higher NT-proBNP. However, it is only when clinical evidence of congestion is confirmed by ultrasound that congestion is associated with a worse outcome. This underscores the non-specific nature of clinical signs of congestion and highlights the need for confirmatory evidence, which can be provided by contemporary ultrasound markers.

Our study highlights the complexity of the relationship between cardiac and renal function. Impaired renal function in patients with HF has been attributed to low cardiac output and renal hypoperfusion.^13^ However, in patients hospitalized with HF, cardiac index does not correlate with renal function, and there is only a weak correlation between right atrial pressure and either creatinine (r = 0.17, *P* = .03) or eGFR (r = −0.20, *P* = .01).^14^ Some authors suggest that raised venous pressure is a key driver of worsening renal function in HF.^4^ However, our findings show that the relation between eGFR and each of the individual markers of congestion using ultrasound is quite weak. The implication is that, in unselected ambulatory patients with chronic heart failure, congestion is not a key determinant of renal function. It follows, then, that other factors (intrinsic renal disease, reno-vascular disease, renal arterial perfusion pressure, diuretic dose or other medications, and comorbidities) influence renal function to a varying extent for individual patients. Other studies also show that clinical and ultrasound signs of congestion are inconsistently associated with a lower eGFR but suggest that it is the presence of congestion that identifies those with the worst prognosis.^15^ We expand on these findings by showing that many patients who are not congested on clinical examination have ultrasound evidence of congestion, which is associated with more severe symptoms and a higher NT-proBNP. Although congestion by ultrasound alone was not statistically associated with an adverse prognosis in the current analysis, this may reflect the small size of the cohort.

Timely identification and management of congestion are challenging. Clinical signs of congestion occur late, and their assessment is subjective and dependent on clinical experience and skill. Ultrasound offers the possibility to identify congestion earlier and objectively in different organs. However, it is not clear what the consequences of discovering congestion on ultrasound alone should be. In a patient with HF in whom there is clinically overt congestion, loop diuretic initiation or intensification is recommended by guidelines. Whether initiating or intensifying loop diuretics in patients with CHF who are congested by ultrasound alone leads to any clinical improvement or change in either natriuretic peptide level or renal function is not known.

Peripheral oedema and exertional dyspnoea are not specific to HF and may result from conditions that mimic or overlap with HF, including venous insufficiency, hypo-albuminemia, obesity, anaemia, and lung disease. Worryingly, loop diuretics, a proxy for congestion, are commonly prescribed without investigations to exclude cardiac dysfunction, leading either to delays in the diagnosis of HF or the diagnosis being missed entirely.^16,17^ In the future, the ready availability of high-quality handheld ultrasound probes powered by artificial intelligence may enable broader and timely access to bedside assessment of cardiac function and congestion, supporting personalized treatment plans for patients with suspected or confirmed HF. However, further research is needed to validate this approach and develop effective treatment strategies.^18,19^

Ultrasound measurements of congestion do have limitations. For instance, IVC assessment can be particularly challenging in obese individuals and requires training and experience. Intravascular volume can also be assessed by evaluating the jugular venous distensibility by ultrasound: an already engorged jugular vein, which shows little changes with Valsalva, suggests right ventricular dysfunction and/or elevated right atrial pressure, and the need for treatment to relieve congestion.^7,9^ Learning how to assess the jugular vein and lungs by ultrasound may be easier and take less time than assessing the IVC. The presence of B-lines can be due to parenchymal lung disease or infection, rather than congestion due to HF.^20,21^

This was a single-centre study conducted within a specialized HF service; external validation in broader and more diverse cohorts would be warranted to confirm the generalisability of these findings. While inter-observer reproducibility was not formally assessed in this study, IVC and JVD ratio assessment has low intra- and inter-observer variability in our experience.^9,22^ Isolated clinical or ultrasound congestion was not associated with a greater number of events compared to the absence of congestion in this analysis. This may be partly explained by the fact that our definition of congestion was based solely on the presence of clinical or ultrasound signs, without accounting for their severity. It is possible that treatments prescribed based on clinical and ultrasound findings, including higher doses of loop diuretics, may have resolved congestion for some and/or worsened renal function and subsequent outcome for others. Additionally, the study may not have been sufficiently powered to detect statistically significant differences for the small group of patients with congestion using only one modality, in whom congestion was less severe. This was an observational study, and renal function and congestion were assessed at a single time point, preventing causal inference and determination of the directionality of the cardiorenal interaction. Moreover, we only measured venous and lung congestion and did not measure congestion in any other tissues or organs.^15^ It might be that congestion in other tissues needs to be managed differently. Different formulae are available to estimate renal function,^23^ and creatinine-based eGFR may be an imprecise measure of true renal function in older patients with HF because of reduced muscle mass, potentially leading to different findings.

## Conclusions

Patients with CHF often have clinical evidence of congestion, confirmed by ultrasound, which is associated with a poor prognosis. Patients with a low eGFR are more likely to be clinically congested, but the relationship between renal function with individual ultrasound measures of congestion is weak. Whether ultrasound assessment of congestion can enhance clinical decision-making in patients with CHF requires further attention and research.

## Supplementary Material

xvag126_Supplementary_Data

## Declarations

### Data Availability

The data that support the findings of this study are available from the corresponding author upon reasonable request and subject to the necessary administrative permissions.

### Pre-registered Clinical Trial Number

None supplied.
