# Supplementary material for: Congestion and renal function in patients with chronic heart failure
Source: ESC Heart Fail. 2026 May 4;13(3):xvag126. doi: 10.1093/eschf/xvag126 (PMC13282901; doi:10.1093/eschf/xvag126)
Supplement: xvag126_Supplementary_Data [file xvag126_supplementary_data.docx]

|  | | | | | | | | |
| --- | --- | --- | --- | --- | --- | --- | --- | --- |
| **Free from clinical congestion (n=203)** | | | | | | | | |
| **eGFR, n** | **<30, (n=10)** | | **30-44, (n=34)** | | **45-59, (n=51)** | | **≥60, (n=108)** | |
|  | **IVC≤2 cm** | **IVC>2 cm** | **IVC≤2 cm** | **IVC>2 cm** | **IVC≤2 cm** | **IVC>2 cm** | **IVC≤2 cm** | **IVC>2 cm** |
| **N (%)** | 6(60%) | 4(40%) | 24(71%) | 10(29%) | 36(70%) | 13(25%) | 74(68%) | 30(27%) |
| **NT-proBNP** | 291, 587, 1024, **1200** 1375, 2282,8040 | 552, 1240, **1371**, 1501, 5955 | 1135  (449, 1960) | 3,370  (1827, 6957) | 849  (350, 1484) | 3,208  (1554, 3912) | 436  (175, 1029) | 1023  (353, 1543) |
|  | **B-lines<14** | **B-lines≥14** | **B-lines<14** | **B-lines≥14** | **B-lines<14** | **B-lines≥14** | **B-lines<14** | **B-lines≥14** |
| **N (%)** | 10 (100%) | NA | 27(79%) | 7(21%) | 35(69%) | 16(31%) | 97(90%) | 11(10%) |
| **NT-proBNP** | 1308  (587, 2282) | No patients | 1408  (394, 2351) | 2476  (1494, 7963) | 1008  (418, 1675) | 1603  (617, 3672) | 481  (228, 1052) | 1289  (366, 2173) |
|  | **JVD-ratio ≥4** | **JVD-ratio <4** | **JVD-ratio ≥4** | **JVD-ratio <4** | **JVD-ratio ≥4** | **JVD-ratio <4** | **JVD-ratio ≥4** | **JVD-ratio <4** |
| **N (%)** | 7(70%) | 3(30%) | 26(76%) | 7(21%) | 31(60%) | 18(35%) | 90(83%) | 10(9%) |
| **NT-proBNP** | 1024  (552, 1501) | 1240, **2282**, 5955 | 1428  (504, 2476) | 2351  (1827, 7963) | 837  (262, 1554) | 2784  (1110, 3912) | 443  (190, 1064) | 1135  (973, 1413) |
| **With clinical congestion (n=139)** | | | | | | | | |
| **eGFR, n** | **<30, (n=19)** | | **30-44, (n=28)** | | **45-59, (n=36)** | | **≥ 60, (n=56)** | |
|  | **IVC≤2 cm** | **IVC>2 cm** | **IVC≤2 cm** | **IVC>2 cm** | **IVC≤2 cm** | **IVC>2 cm** | **IVC≤2 cm** | **IVC>2 cm** |
| **N (%)** | 8(42%) | 11(58%) | 10(36%) | 18(64%) | 8(22%) | 28(78%) | 25(45%) | 30(53%) |
| **NT-proBNP** | 1365  (1061, 2510) | 4779  (3218, 7627) | 1768  (568, 2780) | 2615  (1770, 4200) | 1368  (652, 2164) | 3198  (1920, 5360) | 1079  (512, 2319) | 1820  (1216, 2881) |
|  | **B-lines<14** | **B-lines≥14** | **B-lines<14** | **B-lines≥14** | **B-lines<14** | **B-lines≥14** | **B-lines<14** | **B-lines≥14** |
| **N (%)** | 8(42%) | 11(58%) | 12(42%) | 16(57%) | 14(39%) | 22(61%) | 33(59%) | 23(41%) |
| **NT-proBNP** | 1328  (1061, 2510) | 4779  (3218, 7627) | 1153  (568, 2697) | 3303  (2236, 5405) | 1266  (620, 2299) | 3696  (2216, 7183) | 1079  (475, 1699) | 2782  (1720, 3627) |
|  | **JVD-ratio ≥4** | **JVD-ratio <4** | **JVD-ratio ≥4** | **JVD-ratio <4** | **JVD-ratio ≥4** | **JVD-ratio <4** | **JVD-ratio ≥4** | **JVD-ratio <4** |
| **N (%)** | 5(26%) | 12(63%) | 11(39%) | 12(42%) | 11(30%) | 24(66%) | 27(48%) | 25(44%) |
| **NT-proBNP** | 872, 1302, **1428**, 3017,35000 | 4081  (3092, 7086) | 1769  (804, 3217) | 2699  (2031, 3977) | 1772  (567, 2528) | 2763  (1992, 5360) | 882  (380, 1502) | 2556  (1488, 3002) |

**Table 1 supplementary:** Prevalence of ultrasound signs of congestion and plasma concentrations of NT-proBNP in patients with and without clinical signs of congestion. NT-proBNP is reported as median and IQR if the number of patients is more than 7; otherwise, the individual values are reported along with the median in bold. There was a small amount of missing data, accounting for the number in cells not always adding to 100%.
